# Frontline Science: Shh production and Gli signaling is activated in vivo in lung, enhancing the Th2 response during a murine model of allergic asthma

**DOI:** 10.1189/jlb.3HI1016-438RR

**Published:** 2017-02-24

**Authors:** Ariane S. I. Standing, Diana C. Yánez, Rosie Ross, Tessa Crompton, Anna L. Furmanski

**Affiliations:** * School of Life Sciences, University of Bedfordshire, Luton, UK; and; † Immunobiology Section, UCL Great Ormond Street Institute of Child Health, London, UK

**Keywords:** allergy, morphogen, Gli1, Sonic Hedgehog, epithelia

## Abstract

The pathophysiology of allergic asthma is driven by Th2 immune responses after aeroallergen inhalation. The mechanisms that initiate, potentiate, and regulate airway allergy are incompletely characterized. We have shown that Hh signaling to T cells, via downstream Gli transcription factors, enhances T cell conversion to a Th2 phenotype. In this study, we showed for the first time, to our knowledge, that Gli-dependent transcription is activated in T cells in vivo during murine AAD, a model for the immunopathology of asthma, and that genetic repression of Gli signaling in T cells decreases the differentiation and recruitment of Th2 cells to the lung. T cells were not the only cells that expressed activated Gli during AAD. A substantial proportion of eosinophils and lung epithelial cells, both central mediators of the immunopathology of asthma, also underwent Hh/Gli signaling. Finally, Shh increased *Il-4* expression in eosinophils. We therefore propose that Hh signaling during AAD is complex, involving multiple cell types, signaling in an auto- or paracrine fashion. Improved understanding of the role of this major morphogenetic pathway in asthma may give rise to new drug targets for this chronic condition.

## Abstract (precis)

Hh/Gli signals are received by multiple pulmonary and immune cell types in response to allergen inhalation in vivo; this autocrine/paracrine activation enhances Th2 immune responses.

## Introduction

Asthma is a complex, heterogeneous respiratory disease characterized by airway inflammation, hyperresponsiveness, obstruction, and remodeling. For many, asthma is a chronic, life-long condition controlled by inhaled corticosteroids, leukotriene antagonists, and short- or long-acting bronchodilators. Approximately 3 people per day die as a result of asthma in the UK alone (Asthma UK Data Portal and Office for National Statistics, London, UK). Asthma can be subdivided into a series of variable phenotypes, categorized by the prominent cellular infiltrates and pathophysiology observed in the airway [1]. Most asthma episodes are thought to be triggered by allergens (allergic asthma) and are characterized by eosinophilia and Th2 immune responses. Allergic conditions are linked to epithelial barrier dysfunction, which potentiates immune responses to allergens, although the mechanisms behind this are not clear [2].

Allergic asthma results from misguided immune reaction to aeroallergens, the most common of which are derived from HDMs [3]. Sensitization is complex and is dependent on a series of genetic and environmental factors, including extent of aeroallergen exposure, predisposition to atopy, composition of personal microbiota, exposure to pollutants and time spent indoors in early life [4]. Aeroallergens are ingested by mucosal APCs and presented to T cells in local LNs. T cell activation and conversion to a Th2 phenotype triggers the production of IgE, subsequent activation of mast cells, eosinophils and the production of inflammatory mediators. Persistent allergen exposure, sustained immune infiltration, and chronic inflammation result in excessive mucus production, smooth muscle contraction, airway remodeling, and hyperresponsiveness, leading to the symptoms of asthma.

The mechanisms driving Th2 conversion and persistence in response to inhaled allergens are incompletely characterized. The heterogeneity of asthma phenotypes, disease course, and treatment response implies that there are many factors that synergize to determine phenotype and increase overall risk of disease and treatment failure. It is therefore important to dissect the molecular mechanisms that contribute to the induction and persistence of Th2 immune pathology.

We have shown that Hh signaling to T cells potentiates differentiation of naive T cells to a Th2 phenotype and that Shh ligand is upregulated in the airway of mice with allergic airway disease, a murine model of asthma pathophysiology [5]. This work indicates that Shh is one such factor that can contribute to Th2-mediated responses. The relationship between Shh signaling and allergic asthma has not been explored in detail to date.

Hh proteins are intercellular signaling molecules that are vital for patterning during embryogenesis but also play several roles after birth in tissue homeostasis [6]. There are 3 mammalian Hh proteins: Shh, Dhh, and Ihh, all of which share a common signaling pathway. Upon ligand binding to the Hh receptor Ptch, signal transduction is initiated by derepression of Smo, which triggers an intracellular signaling cascade culminating in the activation of Gli transcription factors. Gli-dependent transcription of target genes is driven by the abundance of GliA relative to transcriptional GliR in the cell. Gli1 acts as an activator of transcription, Gli2 as an activator and repressor, and Gli3 predominantly as a transcriptional repressor [7]. Target genes of Gli are wide ranging and cell-context dependent. We have shown that in mature CD4^+^ T cells, Gli target genes include Hh signaling molecules: *Ptch1* [8], *Smo* [5]; Th2-modulators: *Il-4*, *Il-1rl1* [5]; and genes linked to allergic disease: *Tgfb3* [9], *Igf1r* [10], and *Csf2* [11].

Several genes related to the Hh signaling pathway have been linked to asthma. Large GWASs have implicated *HHIP* [12], *PTCHD1* [13], and *PTCHD3* [14] in poorer asthmatic lung function. Gene expression analyses comparing Th2-high and Th2-low asthma phenotypes found differences in expression of *Dhh* and *Disp1* between groups [15]. These findings have been predominantly discussed in the context of developmental biology, with the authors suggesting that the Hh pathway may be linked to lung function as a consequence of its role in lung branching morphogenesis.

We have shown that Hh signaling via Gli-dependent transcription potentiates the conversion of naive T cells to Th2 effectors by upregulation of Th2-related genes including the key cytokine IL-4 [5]. We therefore propose that the linkage between Hh signaling and asthma may be due to the influence of Hh on Th2 immune responses in addition to any structure–function effects driven by differences in morphogen signaling during lung development and postnatally in lung tissue homeostasis. In this study, we investigated the relationship between Hh/Gli signaling and allergic immune responses in vivo using murine models of asthma pathology.

## MATERIALS AND METHODS

### Mice, tissues, and cells

*Lck*-Gli2ΔC2 (C2, Gli2R [8]), GFP-Gli reporter mice (Gli binding site-GFP transgenic, GBS-GFP Tg [16]), and littermate/age-matched controls, all C57BL/6 background, and C57BL/6 and BALB/c WT mice were bred and maintained at UCL under UK Home Office regulations. AAD was induced as described [5]. HDM allergen was given 3 times per week for the number of weeks stated in the figures. BAL, lung lobes, and draining LNs were harvested. Lung tissue was cryopreserved for sectioning or homogenization or minced and digested with 1.5 mg/ml Liberase (Roche Diagnostics, Burgess Hill, UK) and 0.5 mg/ml DNAse (Roche Diagnostics), subjected to erythrocyte lysis and prepared for flow cytometry, or subjected to lysis for RNA extraction. To obtain SiglecF^+^ cells (eosinophils), cells were purified from pooled BALB/c lung and spleen leukocytes by streptavidin-based magnetic bead positive selection (Thermo Fisher Scientific, Waltham, MA, USA) using anti-SiglecF-biotin (Miltenyi, Bergisch-Gladbach, Germany). Cells were then cultured at 2.5 × 10^5^/ml in AIM V medium (Thermo Fisher Scientific) for 24 h in the presence or absence of 500 ng/ml recombinant Shh (R&D Systems, Minneapolis, MN, USA) before lysis for RNA extraction.

### Flow cytometry and cell sorting

Samples were stained with antibodies from Thermo Fisher Scientific, acquired on a C6 Accuri flow cytometer (BD Biosciences, Franklin Lakes, NJ, USA) and analyzed using FlowJo v10 (Tree Star, Ashland, OR, USA). For cell sorting, samples were acquired on a MoFlo XDP or FACSDiva (Beckman Coulter, Brea, CA, USA) and defined as follows: epithelium, CD45-Epcam^+^; endothelium, CD45-Epcam-CD31^+^; eosinophils, SiglecF^+^CD11b^+^; neutrophils, CD11b^+^Ly6G(IA8)^hi^; and alveolar macrophages, CD11b^lo^CD11c^+^; CD4^+^ T cells, CD4^+^CD11c^−^.

### qPCR

qPCR was performed in triplicate for at least 2 independent experiments with Quantitect primers (Qiagen, Valencia, CA, USA) on a Rotor-Gene thermal cycler (Qiagen) or an iCycler (Bio-Rad, Hercules, CA, USA). Data were normalized to *Hprt* expression and are represented as relative mean expression ± sd of independent experiments, the number of data points indicating the number of mice or samples analyzed.

### Immunofluorescence

Immunofluorescence was performed on fresh frozen acetone-fixed 5 µm sections of OCT-embedded lung tissue. All antibodies were from Thermo Fisher Scientific, unless otherwise stated. To detect Shh: goat anti-Shh clone N19 (Santa Cruz Biotechnology, Dallas, TX, USA), followed by donkey anti-goat biotin (Alpha Diagnostic International, San Antonio, TX, USA) and streptavidin-Alexa Fluor 555 or anti-biotin Alexa Fluor 488; E-cadherin: anti-E-cadherin followed by anti-rat IgG1 PE; SiglecF: anti-mouse SiglecF and anti-rat IgG2a eFluor570; CD16: anti-mouse CD16/32 followed by anti-rat IgG2a eFluor570; CD45: anti-mouse CD45.2-FITC; and CD31: anti-mouse CD31-APC. Data were captured on a BX63 epifluorescence microscope (Olympus, Tokyo, Japan) or an LSM 710 confocal microscope (Zeiss, Jena, Germany) and analyzed using cellSens (Olympus) and Image J (National Institutes of Health, Bethesda, MD, USA) software. Magnifications are given as power of microscope objective; where multiple magnifications are used in a single figure, scale bars are also included.

### Lung histology

Lung lobes were formalin-fixed, paraffin-embedded samples. Sections (5 µm) were subjected to PAS staining with hematoxylin counterstaining, assessed by a blinded observer, and scored for cellular infiltration and PAS^+^ mucus production. Scores denote infiltration and mucus production as 0–1, minimal; 1–2, moderate; and 2–3, severe.

### Data analysis

Statistical analyses were performed with Microsoft Excel or Prism 4 (Graph Pad, San Diego, CA, USA). Two-tailed unpaired Student's *t* tests were used to assess statistical significance, which was accepted at *P* < 0.05. All data are represented as means ± sem, with the exception of qPCR data, which are displayed as stated above.

## RESULTS AND DISCUSSION

### Shh expression increases in lung during AAD induction by allergen administration

We have shown that Shh signals to T cells to favor a Th2 immune response and that Shh protein is increased in the lung tissue of mice after 3 wk of allergen dosing [5]. To further understand when and where Hh ligand is expressed during AAD induction, we examined the expression of Shh in lung during a time course of allergen administration. BALB/c mice were given 3 doses of HDM allergen per week for 0, 1, 2, 3 or 5 wk. A progressive induction of a classic Th2 immune response was observed, including the appearance of CD4^+^T1ST2^+^ (Th2) cells in BAL (Supplemental Fig. S1A), lung (Supplemental Fig. S1B) and eosinophilia (Supplemental Fig. S1C). Concurrently, we observed a progressive increase in Shh mRNA (Supplemental Fig. S1D) and protein (Supplemental Fig. S1E) in lung tissue. Shh colocalized with E-cadherin^+^ airway epithelia (Supplemental Fig. S1F), supporting our published observations. Further, as early as 1 wk into the AAD time course we also observed Shh expression around pulmonary vessels (v) and in areas of cellular infiltration (i) around bronchi/bronchioles (Supplemental Fig. S1F). Shh colocalized with a subset of peribronchiolar CD45^+^ cells (Supplemental Fig. S1G), indicating that in addition to epithelia, cells of hematopoietic origin express Shh during AAD. We therefore show that there are several potential cellular sources of Shh, some of which are recruited to the disease site during AAD.

### Lymphocytes respond to Gli-activating signals in vivo during AAD

**Figure 1.**
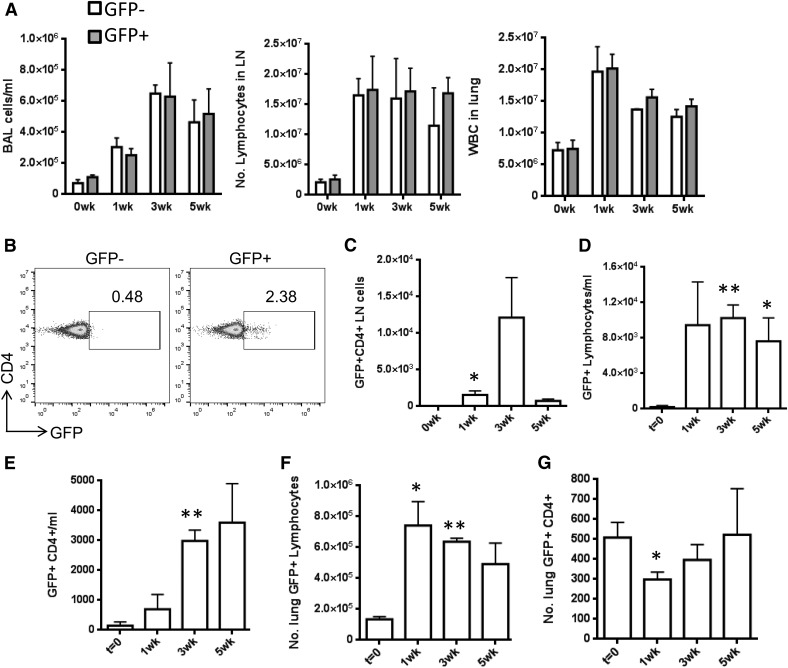
Lymphocytes respond to Hh signaling in vivo during AAD. GFP-Gli reporter mice (GFP-Gli^+^, GFP^+^) and WT (GFP-Gli^−^, GFP^−^) littermates (*n =* 3 per group) underwent repeated intranasal challenge with PBS or 25 μg HDM allergen in PBS. Mean ± sem cell count in BAL, leukocytes in LNs, and lung digests (A) , in GFP-Gli^+^ groups (B, C) GFP status of CD4^+^ cells in LNs (numbers indicate percentage of positive cells). Mean ± sem number of GFP^+^ lymphocytes (D) and CD4^+^ cells (E) in BAL (F) and lung (G), respectively. *P ≤ 0.05; **P ≤ 0.005, compared to the control group at *t* = 0 wks.

To directly test our hypothesis that lymphocytes receive Hh/Gli-activating signals in vivo during AAD [5], we performed a time-course allergen dosing experiment on GFP-Gli reporter mice [16] and WT (non-GFP) littermates. GFP-Gli^+^ mice are engineered to express GFP in cells that have received an activating signal upstream of the Gli transcription factors. GFP expression inside cells reflects the presence of GliA and therefore activation of Gli-dependent transcription. To ensure that the reporter did not have any background effect on our experiments, we compared AAD immune parameters in GFP-Gli^+^ and GFP-Gli^−^ littermates. Across a 5 wk AAD time course, there were no differences in immune cell recruitment between the 2 genotypes, indicating that the presence of the reporter construct does not itself have any influence on AAD progression (**Fig. 1A**).

Using GFP-Gli^+^ mice, we then examined expression of GFP in lymphocytes and CD4^+^ cells during an AAD time-course. GFP^+^CD4^+^ cells were observed in draining LNs after only 1 wk of allergen dosing, with a further increase at 3 wk (Fig. 1B, C). GFP^+^ cells were also evident in BAL (Fig. 1D, E) and lung (Fig. 1F, G), indicating that lymphocytes undergo Gli-dependent transcription during AAD. Upon allergen inhalation, the number of GFP^+^CD4^+^ cells increased in LNs, particularly in BAL, which represents a major site of immune reactivity at the interface between the epithelium and the airspaces. These data suggest that there is a subset of T cells in vivo that are able to respond to Hh or other Gli-activating signals and that these cells increase in number during AAD. Our previous work suggested that such cells would potentiate a Th2 response [5]. We therefore hypothesized that repression of Gli activity in T cells would limit a Th2 response in vivo and dampen allergic pathology.

### Repression of Gli activity in T cells impairs the recruitment of CD4^+^ and Th2 cells during AAD

**Figure 2.**
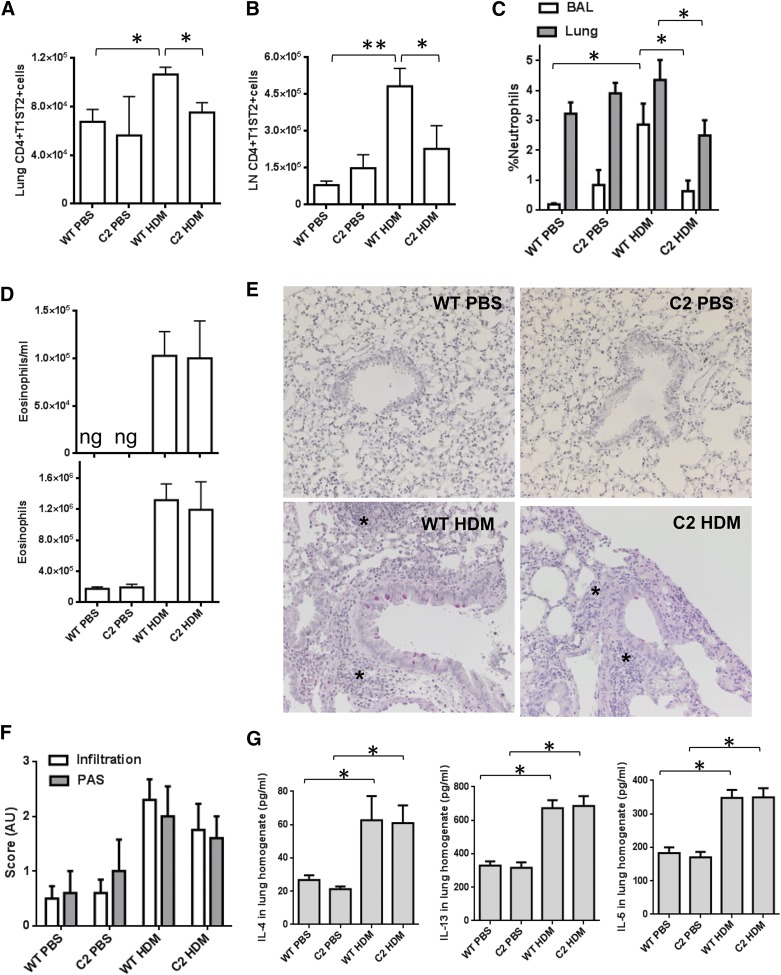
Repression of Gli activation in T cells lowers Th2-cell recruitment and neutrophilia in AAD. C2 (Gli2R) and WT littermates (*n =* 5 or 6 per group) underwent intranasal challenge with PBS or 25 μg HDM allergen in PBS, receiving 9 doses over 3 wk. Flow cytometric analysis of the mean ± sem number of CD4^+^T1ST2^+^ Th2 cells in lung digests (A) and in LNs (B), percentage of CD11b^hi^Ly6G(IA8)^+^CD11c-SiglecF- neutrophils in live lung and BAL leukocyte gates (C), and number of eosinophils in BAL and lung (D). (E) PAS and hematoxylin staining of formalin-fixed, paraffin-embedded lung sections. Asterisks: highly cellular immune infiltrates. Representative examples are shown; magnification ×20. (F) operator-blinded scoring of cellular infiltration and mucus production in PAS/hematoxylin sections: 0–1, minimal; 1–2, moderate; and 2–3, severe. (G) ELISA analysis of cytokine concentration in whole-lung homogenates. *P ≤ 0.05; **P ≤ 0.005.

To test the effect of repressing Gli activity in T cells on AAD, we induced disease in transgenic mice that overexpress the repressor form of Gli2 (Gli2R) under the control of the Lck promoter (C2, Lck-Gli2R). Gli-dependent transcription was therefore repressed in these mice in T cells only: C2 T cells do not efficiently upregulate Gli target genes compared to WT T cells, upon receipt of an Hh signal [8]. C2 and WT littermates were given PBS or HDM allergen for 3 wk and the T cell phenotype was examined. As predicted, significantly fewer CD4^+^T1ST2^+^ Th2 cells were recruited to the lungs (**Fig. 2A**) and LNs (Fig. 2B) of C2 vs. WT mice during AAD. This result indicates a specific effect of Gli2 repression on the differentiation and recruitment of CD4^+^ Th2 cells during AAD. It is particularly noteworthy that C2 tissues contain fewer CD4^+^T1ST2^+^ cells during AAD because these transgenic mice show enhanced selection of CD4^+^ single-positive cells in the thymus and an increased proportion and enhanced signaling, activation, and proliferation potential of CD4^+^ cells in peripheral lymphoid tissues [8, 17].

We noted a significant decrease in the presence of neutrophils in the lung and BAL of C2 mice after allergen administration (Fig 2C). This finding implies that the repression of Gli activity in T cells has a secondary effect on the recruitment, activation, or differentiation of neutrophils in response to HDM inhalation. There was a comparable number of neutrophils in lung and BAL in the PBS-treated C2 and WT groups (Fig 2C), suggesting that neutrophil development is not impaired in C2 mice. It therefore seems plausible that the presence of GliR in T cells alters the production of a factor that controls neutrophil proliferation, mobilization or chemotaxis to the lung in response to inflammation or danger. Of interest, C2 CD4^+^ T cells express lower levels of *Csf2* transcript than WT [5]; this gene encodes GM-CSF, a key cytokine for neutrophil recruitment and activation [18].

However, when we measured AAD pathology in the lungs of C2 and WT mice, we noted no significant differences between the 2 genotypes. Despite the decrease in the number of Th2 cells, eosinophilia was not significantly different (Fig. 2D), PAS staining revealed comparable mucus production, and histologic examination of lung infiltration suggested that overall, AAD pathology was not diminished in the lungs (Fig. 2E, F). Th2-related cytokines in whole-lung homogenates were equivalently induced by HDM treatment in WT and C2 groups (Fig. 2G), despite the fact that the T1ST2^+^ population was reduced in C2 mice. This result suggests that other cells are responsible for most IL-4, -5, and -13 in lung. Indeed, Th2 cells are not the only source of these cytokines: among other cells, eosinophils, recruited in large numbers during AAD, also produce IL-4, -5, and -13 [19].

We therefore considered the possibility that there are other, as yet uncharacterized cells in the lung that respond to Shh during AAD and influence Th2 responses.

### Innate immune cells and lung epithelium respond to Gli-activating signals in vivo

**Figure 3.**
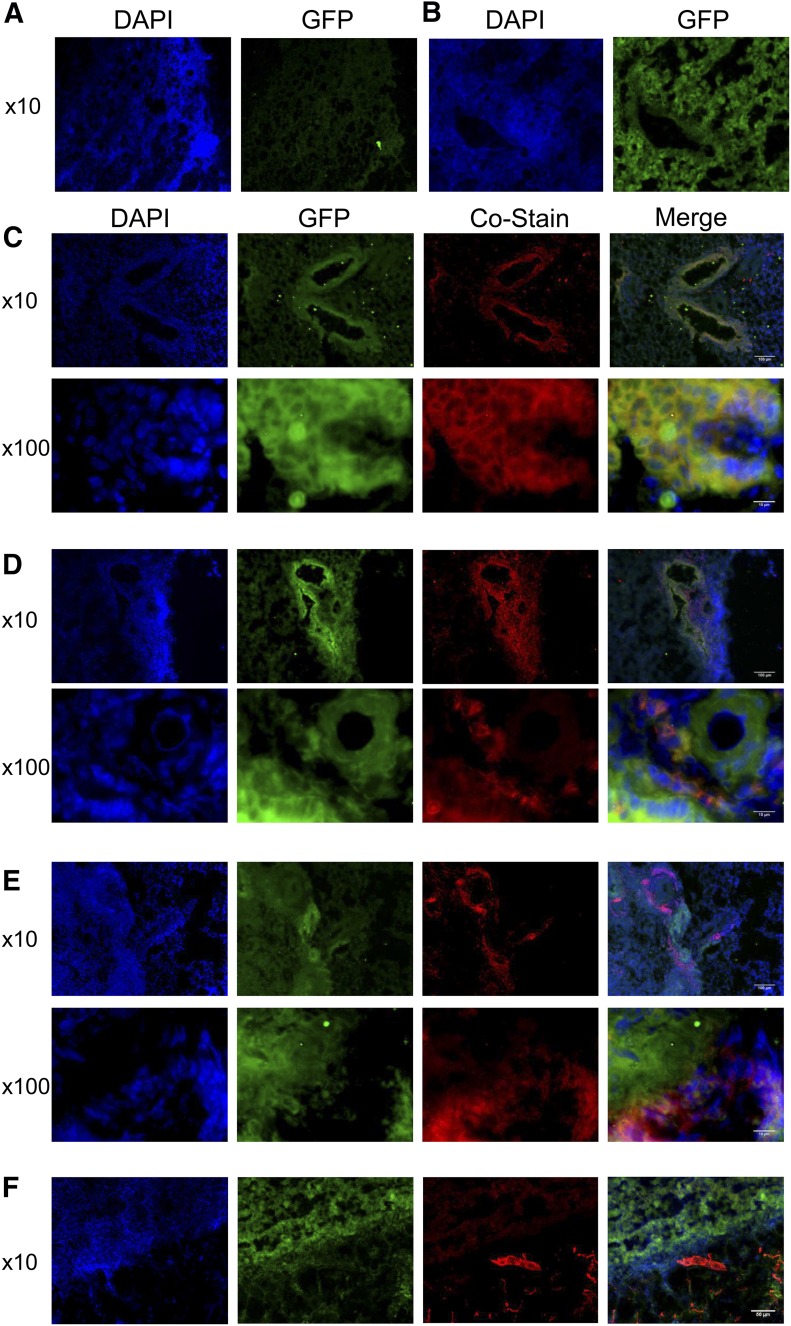
GFP-Gli is expressed in epithelial cells and subsets of innate leukocytes. GFP expression in GFP-Gli- control (A) and in GFP-Gli^+^ (B) lung cryosections. Immunofluorescence of GFP-Gli^+^ lung cryosections after 3 wk allergen inhalation following costaining with anti-E-cadherin(C), anti-SiglecF (D), anti-CD16 (E), and anti-CD31(F). Colocalization in red/green merged images appears as yellow or paler pink, depending on the intensity of the green fluorescence.

To assess whether other cells involved in AAD respond to Hh/Gli signaling, we screened for GFP expression and thus GliA during an AAD time course in GFP-Gli^+^ reporter mice. We examined sections of lung lobes to determine the location of GFP^+^ cells. We found no GFP expression in GFP-Gli^−^ littermate lung samples (**Fig. 3A**), but GFP was highly expressed around lung structures including bronchial/bronchiolar and alveolar epithelium and diffusely throughout other areas of the lung during AAD in GFP-Gli^+^ lungs (Fig. 3B). We noted variable and graded expression of GFP throughout the tissue, implying that some cells expressed higher levels of Gli than others. This finding is consistent with the graded signaling that would be expected with a morphogen and is detectable with this reporter, which has been used to model the dynamics of morphogen signaling in neural development [16]. It is currently unknown to what extent morphogen gradients function in adult tissue. Hh signaling in adult lung has promoted pro mitogenic [20, 21] and antiproliferative [22] effects in models of acute lung injury. These conflicting results could be related to differences in experimental models or may reflect functional differences in morphogen gradients in tissues.

Coimmunofluorescence analyses showed that GFP signal was strongest in E-cadherin^+^ bronchial cells and in peribronchial cells after 3 wk allergen inhalation, particularly at the air interface (Fig. 3C). Weaker GFP signal (seen on merged images as pink-yellow, depending on the strength of the GFP signal) also colocalized with a subset of peribronchial SiglecF^hi^ cells—possibly the eosinophils about to egress into the airspaces (and therefore BAL)—after HDM dosing (Fig. 3D). Colocalization of GFP was also observed with some CD16^+^ cells; thus, we also detected Gli activity in a subset of phagocytic cells (Fig. 3E). We did not detect GFP expression in CD31^+^ cells at 3 wk (Fig. 3F), indicating that pulmonary endothelium does not express active Gli in AAD at this time point. These data indicate that lung epithelial cells, some eosinophils, and phagocytes are all subject to Hh/Gli signaling during AAD.

**Figure 4.**
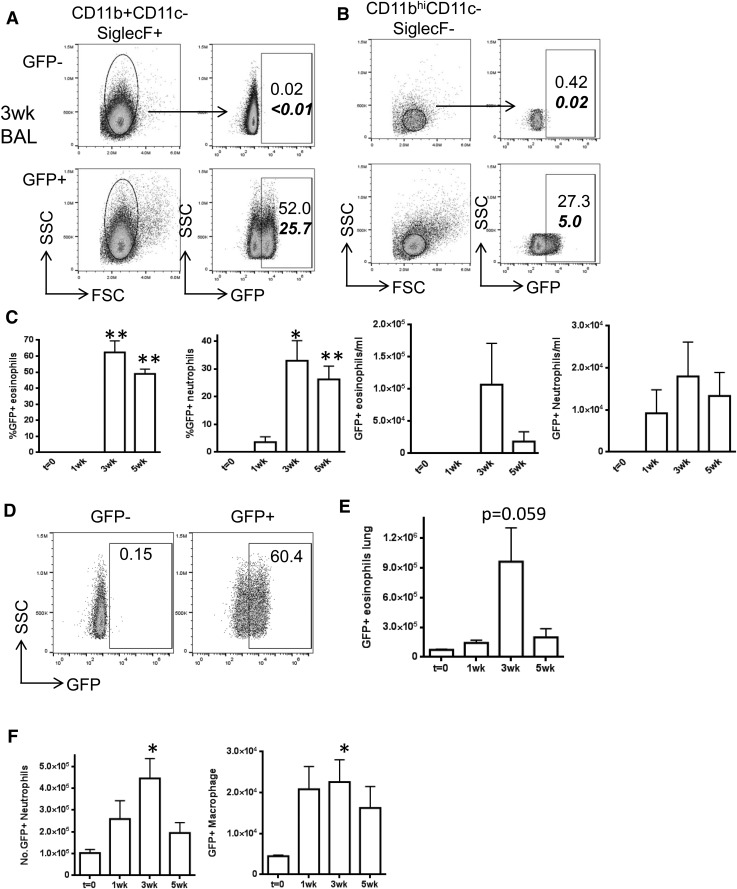
Innate immune cells express can respond to Hh/Gli signals during AAD in vivo. GFP-Gli reporter mice (*n =* 3 in each group) underwent repeated intranasal challenge with 25 μg HDM allergen in PBS as described. Flow cytometry was used to analyze eosinophils (A) and neutrophils (B) in BAL. Representative flow profiles are shown at 3 wk; numbers indicate percentage of eosinophils, bold numbers indicate percentage of live gate. (C) Percentage and number per ml BAL of GFP^+^ eosinophils and neutrophils, (D) %GFP^+^ in lung CD4^+^ T cell populations and number of GFP^+^ lung (E) eosinophils, (F) neutrophils and macrophages. *P ≤ 0.05; **P ≤ 0.005, compared to the control group at *t* = 0 wks.

We also quantified cells expressing GliA by flow cytometry in whole lung homogenates and in BAL. At 3 wk most of the eosinophils in BAL expressed GFP (**Fig. 4A**), as did a lower but still substantial proportion of neutrophils (Fig. 4B). GFP expression was sustained in these cells into wk 5 of allergen dosing (Fig. 4C). GFP expression was also observed in a proportion of lung eosinophils (Fig. 4D, E), in a minority of lung neutrophils and a subset of lung macrophages (Fig. 4F). These data indicate that innate immune cells, in particular a significant subset of eosinophils, are capable of responding to Hh or other Gli-activating signals during allergen inhalation. To our knowledge, this has never been reported. We noted a much higher frequency of GFP^+^ eosinophils in BAL and lung digests when we analyzed samples from our AAD experiments by flow cytometry (Fig. 4), compared with immunofluorescence (Fig. 3). This effect may be caused by the enhanced sensitivity of cytometry or by cytometric gating strategies, and lung cryosections, which are extensively washed during staining, may simply not mimic the cellular content of intact whole-lung digests. Alternatively, it may represent a real difference in Gli activation. BAL contains eosinophils that have egressed from pulmonary tissue, which may well have received Hh signals when passing through the epithelial barrier. Lung and BAL eosinophils are also functionally distinct during AAD, with inflammatory eosinophils located in BAL and peribronchial areas and regulatory eosinophils found largely in lung parenchyma [23]. The function of eosinophils can thus be heavily influenced by their location in tissue, presumably via responses to local microenvironmental signals; we propose that Shh/Gli signaling is one such local signal, active during allergic responses.

### Lung epithelium, endothelium, and innate immune cells, particularly eosinophils, express key molecules of the Hh signaling pathway

**Figure 5.**
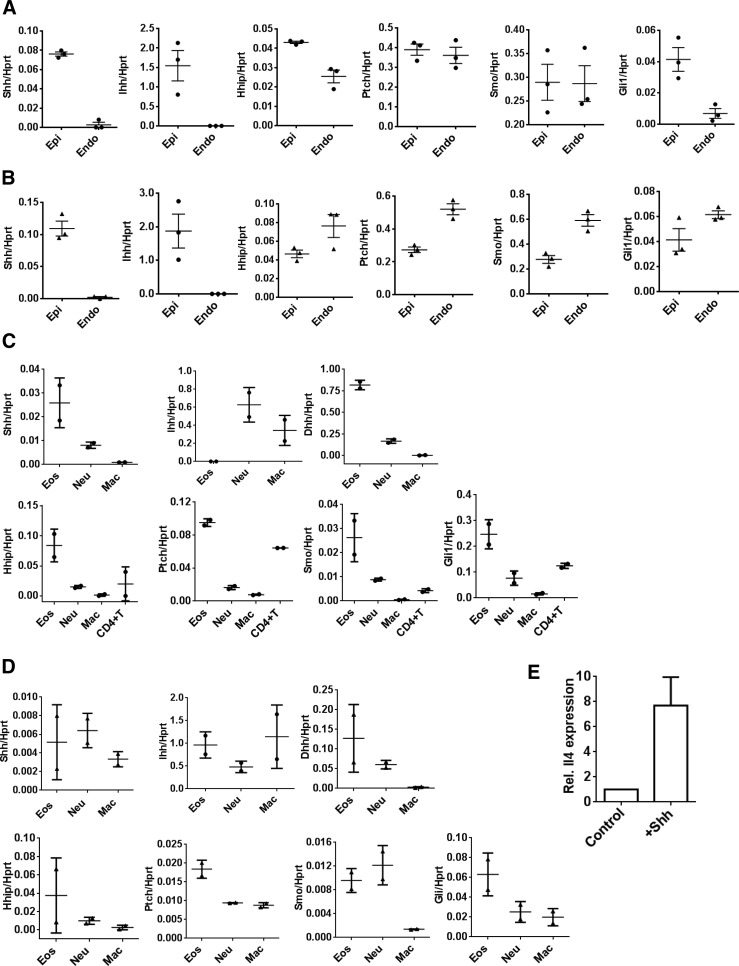
Innate immune cells, particularly eosinophils, express key molecules of the Hh signaling pathway. Lungs were harvested from C57BL/6 (A, B; *n =* 3 per group) or BALB/c WT (C, D; *n =* 2 per group) allergen-naive (A, C) or HDM-treated (B, D) mice and lung epithelial and endothelial (A, B) or resident (C, D) lung eosinophils, neutrophils, macrophages, and CD4^+^ T cells were sorted by FACS and subjected to gene expression analysis by qPCR. Expression of *Shh*, *Hhip*, *Ptch*, *Smo*, *Gli1*, *Ihh*, and *Dhh* are expressed relative to *Hprt* in each cell subset, data points represent individual mice. (E) SiglecF^+^ cells were separated from WT mice (*n =* 2 independent experiments) and cultured for 24 h in the presence (^+^Shh) or absence (control) of 500 ng/ml recombinant mouse Shh protein. Expression of *Il-4* in the treated group was assessed by qPCR in triplicate, relative to the control samples.

To confirm that lung tissue and innate immune cells express the machinery required for Hh/Gli signaling, we sorted epithelial and endothelial cells, eosinophils, neutrophils, macrophages, and CD4^+^ T cells from lung digests of WT mice and performed qPCR to assess gene expression. In control mice, epithelial cells expressed *Shh* (as in ref. 5), *Ihh*, *Hhip*, *Ptch*, *Smo*, and *Gli1* indicating potential to both produce Hh ligand and receive Hh signals (**Fig. 5A**). Endothelial cells expressed negligible levels of *Shh* but did express Hh signaling machinery. Upon HDM inhalation, epithelial cells expressed all of the genes listed above. Compared with epithelial cells, endothelial cells expressed slightly higher levels of *Hhip*, *Ptch*, *Smo*, and *Gli1* (Fig. 5B). These results indicate that the Hh signaling capacity in the endothelial cells of the pulmonary vasculature may change from their baseline state after allergen exposure.

The respiratory epithelium is an important physical barrier and immune organ, increasingly linked to the initiation and maintenance of unwanted allergic immune responses. HDM allergens including Derp1 protease disrupt epithelial tight junctions [24], permitting transepithelial passage of allergen and immune priming. Derp1 also stimulates the release of proinflammatory cytokines from respiratory epithelium [25], which are important in subsequent immune skewing. We show, for the first time to our knowledge, that lung epithelia also express Hh signaling machinery during AAD, suggesting that auto- and paracrine Hh signaling is possible in this compartment after allergen inhalation. Embryogenic morphogens such as Shh are re-expressed in adult lung to maintain epithelial and mesenchymal cell quiescence [22]. Dysregulation of this signaling is strongly implicated in pulmonary fibrosis in mouse models [20] and humans [26]. It is plausible that Shh signaling in epithelial cells contributes to the long-term fibrotic airway remodeling seen in chronic asthma. Hh signaling regulates the EMT during lung development and branching morphogenesis. Asthma has been proposed as a disorder of dysregulated EMT, although this theory is controversial. Nevertheless, morphogenetic pathways appear to be good candidates for driving EMT during lung disease, including asthma (reviewed in ref. 27).

Naive (Fig. 5C) and allergen-exposed (Fig. 5D) eosinophils expressed *Ptch* and *Smo* Hh receptor and signal-transduction molecules, respectively, and *Gli1* at higher levels than any of the other innate immune cells and, indeed, higher than CD4^+^ T cells. Naive eosinophils also expressed Hh ligands, *Shh* and *Dhh*, and *Hhip* (Fig. 5C). These cells are recruited in abundance to BAL (hundreds of thousands) and lung (millions) during the induction of AAD (Supplemental Fig. S1C) and thus are likely to be a major source of Hh ligand. After allergen treatment, *Ihh* expression was unexpectedly induced in eosinophils (Fig. 5D). There is little literature on this response, although Ihh has a role in thymocyte development [28] and macrophage polarization [29]. It is important to fully characterize this and the role of Hh signaling in other innate immune cells before Hh inhibitors are investigated as a potential treatment for Th2-mediated disease. Neutrophils and macrophages from HDM-exposed mice expressed the Hh signal-transduction molecules *Ptch* and *Smo*, but at lower levels than expressed by eosinophils (Fig. 5D). As in baseline conditions (Fig. 5C), neutrophils continued to express Hh ligands. As neutrophil recruitment and/or activation is affected by repression of Gli-dependent transcription in T cells (Fig. 2C), and neutrophils are Gli responsive (Fig. 4), it would be interesting to examine the role of Hh/Gli signaling in neutrophilic/Th17 asthma. Macrophages expressed *Shh* and *Ihh*, but not *Dhh*, after HDM inhalation (Fig. 5D). Shh/Gli ligand expression and signaling have been reported in macrophages/monocytes [29–31]. There is evidence that macrophages upregulate both Shh and Ihh after exposure to schistosome antigens, and that this effect can lead to M2 polarization and a subsequent profibrotic environment in the liver [29]. It remains to be seen whether Hh signaling in lung during AAD favors M2 differentiation and pulmonary fibrosis or airway remodeling.

HHIP is expressed in adult lung tissue including the lung epithelium, pulmonary endothelium, and eosinophils (Fig. 5). Several GWASs have repeatedly identified SNPs at the HHIP locus, the endogenous inhibitor of Hedgehog ligands, to affect lung function [32–34] and to predispose to increased susceptibility to asthma [12]. Patients with chronic obstructive pulmonary disease have decreased HHIP mRNA and protein expression [35]. Functionally, two of the associated SNPs have been shown to be within an HHIP enhancer region that interacts with the HHIP promotor, and both SNPs reduce promotor activity [35]. Altered HHIP levels may well lead to enhanced Shh signaling, and we speculate consequent similar corollaries as we have reported here.

Shh activates Gli-dependent transcription via the canonical pathway. We have established that the components of the canonical hedgehog pathway are present in lung tissue and immune cells. However, we also acknowledge that Hh ligand can signal noncanonically and that Gli-dependent transcription can be triggered by non-Hh ligands, such as TGF-β [36]. The crosstalk of these signaling pathways in AAD and asthma therefore warrants further investigation.

### Eosinophils, recruited to lung and BAL in a high number in AAD, can respond to Hh signals by upregulating transcription of *Il-4*

Our data suggest that eosinophils, key cells in the Th2 response, produce Hh ligands and respond to Hh signals. To directly test the effect of Shh signaling to eosinophils, we purified SiglecF^+^ cells from murine lung and spleen and cultured the cells in the presence or absence of recombinant mouse Shh. As in CD4^+^ T cells [5], Shh treatment triggered upregulation of *Il-4* in eosinophils (Fig. 5E). This finding suggests that Hh ligands, produced by various lung cell types during AAD can further potentiate Th2 cytokine production, and it could explain why repression of Gli activity in T cells only (Lck-Gli2R transgenic) did not significantly alter total IL-4 concentration in AAD lung tissue (Fig. 2C). However, the Smo inhibitor cyclopamine promotes eosinophilic differentiation of leukemic myeloid cells [37]; thus, it is currently unclear what role Hh signaling plays in eosinophil differentiation and function.

**Figure 6.**
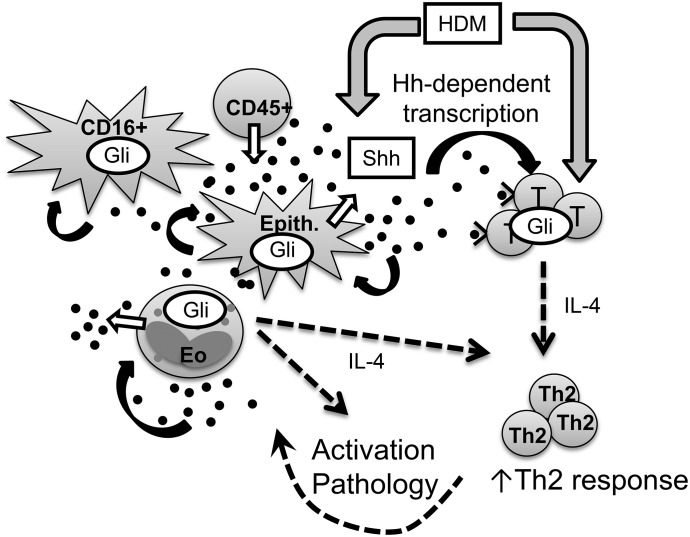
Multiple cell types in lung produce and respond to Shh, potentiating Th2 responses as a consequence of allergen inhalation in AAD. Our working model [**5**] has been updated to incorporate our new findings. We propose that in addition to triggering allergic immune responses via CD4^+^ Th2 activation, HDM allergen also induces the release of Shh in lung (HDM action). Shh is the primary Hh ligand secreted in the lung during AAD (Shh ligand, black circles) and that the source cells include epithelia, eosinophils, and other CD45^+^ leukocytes (Shh release, white arrows). T cells are able to respond to Shh (Shh/Gli response, black arrows), leading to Gli activation and upregulation of genes involved in Th2 skewing, leading to the potentiation of Th2 pathology and further inflammation/injury, potentially further increasing Shh expression. Epithelial cells and some CD16^+^ cells express active Gli (black dashed arrows), likely in response to Shh, but the outcome of the signal to these cell types is currently unknown. Eosinophils also express activated Gli, and one consequence of this via Shh signaling is the upregulation of IL4, which may further drive Th2 activation and disease.

Our initial model proposing that Shh is released from lung epithelial cells after allergen inhalation therefore requires modification to include CD45^+^ innate leukocytes such as eosinophils and neutrophils (**Fig. 6**). The model should also be extended to reflect the Hh/Gli signaling potential of eosinophils, CD16^+^, and epithelial cell subsets, and the influence of Shh on *Il-4* transcription in eosinophils, potentially exacerbating the local Th2 response. The full consequences of Gli activation in each of the cell types examined in this study are currently unknown. In addition to its roles in maintaining quiescence and tissue homeostasis, postnatal re-expression of Shh in lung may therefore also have evolved as a beneficial Th2-potentiating mechanism to protect against lung parasites. Of note, Shh production is increased in astrocytes of mice infected with *Angiostrongylus cantonensis*, the rat lungworm [38]. However, in allergy, excess Shh in lung tissue is potentially pathologic, and it may exacerbate Th2-driven disease. This possibility requires further investigation to fully characterize the role of Hh signaling in the allergic lung, which is clearly complex. Of further interest are group 2 innate lymphoid cells, which lack antigen receptors but are early producers of Th2 cytokines in immune responses. These cells are particularly important in driving Th2 responses in the papain-induced AAD model [39]. Their development is regulated by the morphogenic pathways NOTCH and Wnt/β-catenin (reviewed in ref. 40); thus, it is important to establish whether there is a role for Hh/Gli signaling in the differentiation and function of this cell type.

In summary, this study reports a novel role for Hh signaling in the crosstalk between pulmonary cells and infiltrating leukocytes during the induction of an allergic immune response. Future work should determine the role of canonical and noncanonical Hh/Gli signaling in eosinophils, neutrophils, and airway epithelia in the presence and absence of allergens. The Hh/Gli signaling pathway is complex and subject to several regulatory and feedback mechanisms. It will be very interesting to dissect the kinetics of Hh/Gli signaling in these cell types during AAD. It is not currently clear in which cell type, if any, Hh signaling is absolutely required for AAD induction. It may well be the case that Hh signaling is another pathway that contributes to, rather than controls, disease pathogenesis. Together, this information should uncover novel biologic mechanisms and allow the rational design of potential modulators of Th2-driven lung disease.

## AUTHORSHIP

A.S.I.S. designed and performed the experiments, analyzed the results, and drafted the paper; D.Y.M. and R.R. performed experiments; T.C. contributed to the conception of the study, provided valuable insight, and critically revised the paper; A.L.F. conceived the study, designed and performed the experiments, analyzed and interpreted the results, and wrote the paper.

## Supplementary Material

Supplemental Data

## Abbreviations

AAD: allergic airways disease
BAL: bronchoalveolar lavage
Dhh: Desert hedgehog
EMT: epithelial to mesenchymal transition
Gli: glioma-associated oncogene
GliA: transcriptional activator Gli
GliR: transcriptional repressor Gli
GWAS: genome-wide association studies
HDM: house dust mite
Hh: Hedgehog
Hhip: Hedgehog interacting protein
Ihh: Indian hedgehog
LN: lymph node
PAS: Periodic Acid Schiff
Ptch: patched
Shh: Sonic Hedgehog
Smo: smoothened
SNP: single-nucleotide polymorphism
