# Supplementary material for: Frontline Science: Shh production and Gli signaling is activated in vivo in lung, enhancing the Th2 response during a murine model of allergic asthma
Source: J Leukoc Biol. 2017 Feb 24;102(4):965–76. doi: 10.1189/jlb.3HI1016-438RR (PMC5597515; doi:10.1189/jlb.3HI1016-438RR)
Supplement: Supplemental Data [file supp_102_4_965__index.html]

Frontline Science: Shh production and Gli signaling is activated in vivo in lung, enhancing the Th2 response during a murine model of allergic asthma — Frontline Science: Shh production and Gli signaling is activated in vivo in lung, enhancing the Th2 response during a murine model of allergic asthma — Supplemental Data 

# Frontline Science: Shh production and Gli signaling is activated in vivo in lung, enhancing the Th2 response during a murine model of allergic asthma

## Supplemental Data

- Supplemental Data
- Supplemental Data
