# Supplementary material for: Frontline Science: Shh production and Gli signaling is activated in vivo in lung, enhancing the Th2 response during a murine model of allergic asthma
Source: J Leukoc Biol. 2017 Feb 24;102(4):965–76. doi: 10.1189/jlb.3HI1016-438RR (PMC5597515; doi:10.1189/jlb.3HI1016-438RR)
Supplement: Supplemental Data [file supp_jlb.3HI1016-438RR_Supplemental_Data.docx]

*Supplementary Figure 1: Shh is expressed by lung epithelium and hematopoietic cells during allergen-induced airways disease*

BALB/c WT mice (n=3 in each group) underwent repeated intranasal challenge with 25μg HDM allergen in PBS as described. Flow cytometric analysis of (A) number of T1ST2+CD4+ Th2 cells per ml BAL and in (B) lung digests, and (C) number of SiglecF^+^CD11b^+^FSC^lo^SSC^int^ eosinophils in lung. Statistically significant differences between group and the control group (t=0, no HDM doses) mean±SEM were identified by unpaired t-testing (*p≤0.05; **p≤0.005), ng = negligible. Expression of (D) *Shh* in lung homogenates during AAD by qPCR relative to *Hprt* expression, data points show individual mice, each representing mean of triplicate results (*p≤0.05, vs. control group at t=0 wks; †p≤0.05, vs. group at 1wk; #p≤0.05, vs. group at 2wk). (E) Shh protein measured by ELISA in AAD lung homogenate supernatants (*p≤0.05, compared to the control group at t=0 wks). (F) Immunofluorescent staining of cryosections from AAD time course lungs to assess expression of (F) Shh (green) and E-cadherin (red, epithelial marker), I: infiltrate, V: vessel or vascular structure, and (G) CD45 (green) and Shh (red). Merge (pink/yellow) shows DAPI+ cells and co-localization of Shh and co-immunostain, magnification x20, representative examples shown.
